# Experimental Models to Study Autism Spectrum Disorders: hiPSCs, Rodents and Zebrafish

**DOI:** 10.3390/genes11111376

**Published:** 2020-11-20

**Authors:** Alba Pensado-López, Sara Veiga-Rúa, Ángel Carracedo, Catarina Allegue, Laura Sánchez

**Affiliations:** 1 Department of Zoology, Genetics and Physical Anthropology, Universidade de Santiago de Compostela, Campus de Lugo, 27002 Lugo, Spain; alba.pensado.lopez@rai.usc.es (A.P.-L.); sara.veiga.rua@rai.usc.es (S.V.-R.); 2 Genomic Medicine Group, Center for Research in Molecular Medicine and Chronic Diseases (CiMUS), Universidade de Santiago de Compostela, 15706 Santiago de Compostela, Spain; angel.carracedo@usc.es; 3 Centro de Investigación Biomédica en Red de Enfermedades Raras (CIBERER), CIMUS, Universidade de Santiago de Compostela, 15706 Santiago de Compostela, Spain

**Keywords:** autism spectrum disorders (ASD), animal models, cellular models, genome editing, human induced pluripotent stem cells (hiPSCs), neurodevelopmental disorders (NDDs), rodents, zebrafish

## Abstract

Autism Spectrum Disorders (ASD) affect around 1.5% of the global population, which manifest alterations in communication and socialization, as well as repetitive behaviors or restricted interests. ASD is a complex disorder with known environmental and genetic contributors; however, ASD etiology is far from being clear. In the past decades, many efforts have been put into developing new models to study ASD, both in vitro and in vivo. These models have a lot of potential to help to validate some of the previously associated risk factors to the development of the disorder, and to test new potential therapies that help to alleviate ASD symptoms. The present review is focused on the recent advances towards the generation of models for the study of ASD, which would be a useful tool to decipher the bases of the disorder, as well as to conduct drug screenings that hopefully lead to the identification of useful compounds to help patients deal with the symptoms of ASD.

## 1. Introduction

### 1.1. Definition and Epidemiology of Autism Spectrum Disorders

Autism Spectrum Disorders (ASD)-affected individuals are characterized by the presence of social and communication impairments and the lack of common skills in developing, maintaining, and understanding relationships. In addition to these symptoms, patients might also develop stereotyped or repetitive patterns of behavior, interests and/or activities. According to the 5th edition of the Diagnostic and Statistical Manual of Mental Disorders (DSM-5), the ASD category includes the following neurodevelopmental disorders (NDDs): early infantile autism, childhood autism, Kanner’s autism, high functioning autism, atypical autism, pervasive developmental disorder not otherwise specified (PDD-NOS), childhood disintegrative disorder, and Asperger’s disorder [1].

**Figure 1:**
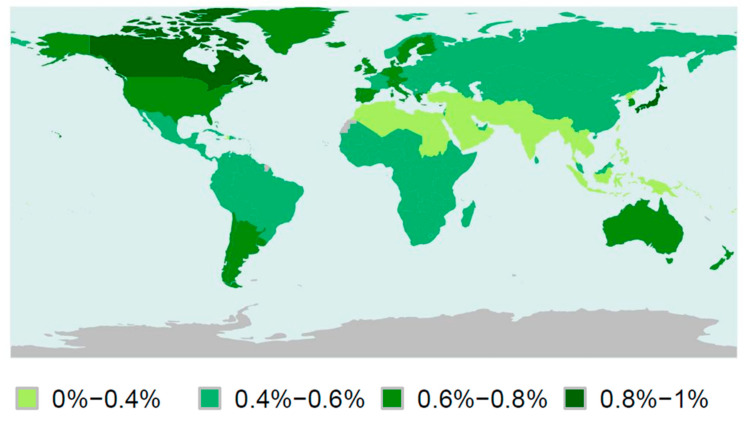
Map of the prevalence of Autism Spectrum Disorders (ASD) around the world in 2017. Light green: prevalence between 0–0.4%; blue: prevalence between 0.4–0.6%; green: prevalence between 0.6–0.8%; dark green: prevalence between 0.8–1%. Countries from which no data are available are plotted in grey. The figure was elaborated using R software (R Core Team, Vienna, Austria) to represent open access data which have been previously standardized to age and sex [2,4,6].

The prevalence of ASD is estimated to be around 1.5% [2,3,4], although these data vary depending on the year and the country dataset consulted (Figure 1). Differences among datasets could be associated with real differences on ASD prevalence, but also with errors due to diagnostic difficulties or lack of trustworthy data [5].

### 1.2. Aetiology of Autism Spectrum Disorders

Depending on whether the origin of ASD is known or not, the disorder can be classified into two subgroups: syndromic and non-syndromic ASD. Syndromic ASD includes those cases with a well-characterized etiology, whereas non-syndromic ASD cases have a less defined etiology, with multiple factors contributing to the development of the disorder [7].

ASD can be linked to prenatal, perinatal and postnatal risk factors, which can be either genetic or environmental [8]. Several environmental factors have been found strongly correlated with ASD development, such as advanced parental age, pregnancy and birth complications, vitamin D deficiency and heavy metal exposition [8,9,10].

Regarding genetics, their relevance in ASD risk development has been known for over 50 years, mainly due to the results observed in twin studies. The first twin studies indicated that ASD concordance could be around ~90% in monozygotic twins, in comparison with a 30% concordance observed in dizygotic twins [11,12,13]. However, recent data seem to indicate that ASD concordance in monozygotic twins might be lower (~50%) [10].

Despite the obvious challenges associated with the identification of ASD causes, many susceptibility genes have been identified by genetic analysis, including exome sequencing and genome-wide association studies (GWAS). ASD-associated genes are frequently involved in the regulation of neural and synaptic development and its alteration can lead to dysfunctions in brain areas that regulate high cognitive functions [13,14,15,16]. In addition, molecular alterations in excitatory cortical neurons, microglia and cortico-cortical projection neurons have also been associated with ASD severity [17].

Both common and rare genetic variants have been associated with ASD development. Available data suggest that de novo mutations in coding regions are among the most frequent variants associated with ASD. However, other genetic alterations such as copy number variations (CNVs) and chromosomal alterations have also been associated with the development of the disorder [7,13,18,19].

One of the most complete recompilation of ASD-associated genes is the SFARI Gene Database [20,21]. In the 2020 database release, genes are classified according to a gene score (1, 2 or 3) that takes into account the amount of information supporting the implication of a certain gene in ASD development. Genes with score 1 are high confidence ASD-associated genes with a minimum of three de novo disrupting mutations linked in patients to the development of the disorder. Genes with score 2 are strong candidates with two de novo disrupting mutations associated with ASD development. Finally, genes with score 3 are those with one reported de novo disrupting mutation linked to ASD, but the results have not been replicated yet.

**Figure 2:**
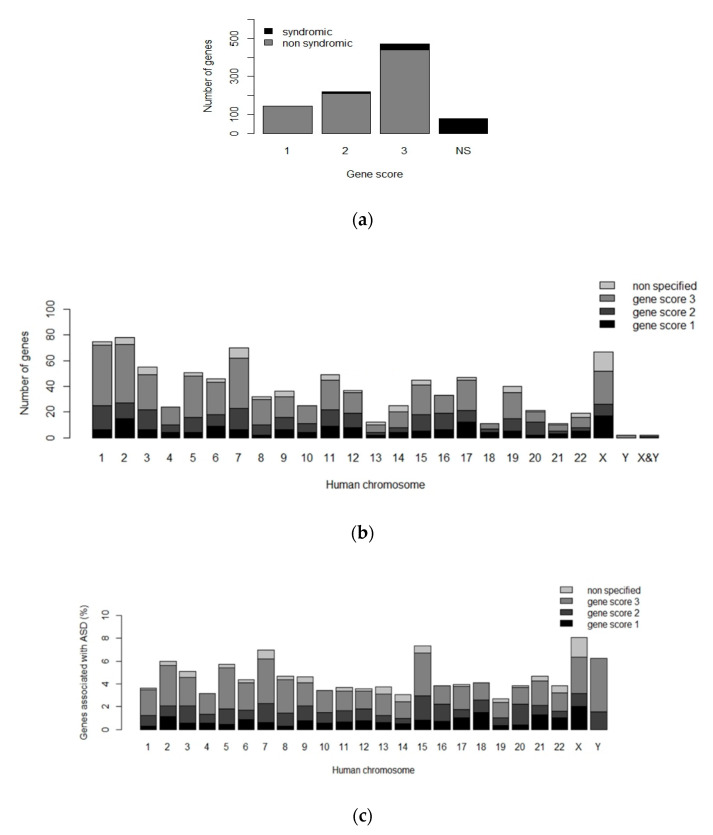
Human ASD-associated genes according to the SFARI Gene Database (2020). Gene score 1: high confidence genes with a minimum of three de novo likely gene disrupting mutations associated to ASD. Gene score 2: strong candidate genes with two de novo gene-disrupting mutations associated to ASD. Gene score 3: suggestive evidence of the association of the gene with ASD development, due to one reported de novo likely gene-disrupting mutation. (**a**) Classification of the 913 ASD-associated genes in the SFARI Gene Database according to the gene score and their presence in syndromic or non-syndromic ASD patients (NS = non-specified); (**b**) ASD-associated genes distribution in the human genome; (**c**) Percentage of ASD-associated genes identified on each human chromosome. The figure was elaborated using open-access data from SFARI Gene Database (obtained in January 2020) and R software [6,20,21].

A total of 913 genes have been registered into the SFARI Gene Database (https://gene.sfari.org/, latest release 2020) as ASD-associated genes with their corresponding score following the previously mentioned criteria (Figure 2a) [20,21]. These genes are not evenly distributed throughout the genome, for instance, high confidence ASD-associated genes (gene score 1) are particularly abundant in the chromosome X (Figure 2b,c). Some authors have linked this observation with the male-to-female ASD ratio which is about 4 to 1 [11,22].

### 1.3. Diagnostic of Autism Spectrum Disorders

Nowadays, ASD diagnosis is based on standard clinical criteria (Table 1) that evaluate the symptoms and their severity in each case [1]. However, ASD symptoms can vary a lot between individuals. In the most severe cases, an accurate diagnosis is usually made at an early age (1–2% of the population), but milder phenotypes can be harder to identify for clinicians, as different NDDs can co-occur and symptoms might be very similar [1,7,23].

The current approach to diagnose and treat ASD patients is far from optimal. To improve this situation, it is essential to broaden the current knowledge of ASD bases, which could give us new insights to improve the diagnosis and treatment of patients.

### 1.4. Treatment of Autism Spectrum Disorders

Treatment for ASD patients is essentially focused on ameliorating the symptoms of the disorder to reduce the impact it has on the daily activities of the affected individuals. To this end, it is frequent that patients receive a combination of therapeutic approaches, including behavioral therapy and/or medication (see Table 2 for a list of ASD-related therapies). There is no medication that can completely alleviate ASD symptoms or cure the disorder. However, some compounds—such as α2-adrenergic agonists and olanzapine—have been approved to ameliorate some symptoms of the disorder, but their efficiency is limited [24,25].

## 2. Genome Editing Systems, a Promising Tool for Modeling Human Disorders

As mentioned before, both genetic and environmental risk factors contribute to ASD development. Due to this complexity, deciphering the individual impact of each risk factor on the development of ASD was a difficult task for researchers for a long time, and it is still a challenge.

This scenario recently changed due to the development of improved genetic edition systems which allow simplifying the study of the function of selected genes and their relationship with disease-related phenotypes. To date, there are three main types of genetic editing systems available: Zinc Finger Nucleases (ZFNs), Transcription Activator-Like Effector Nucleases (TALENs) and CRISPR/Cas (Clustered Regularly Interspaced Short Palindromic Repeats). The first editing tools available were ZFNs, in 1996 [26] and TALENs, in 2010 [27], both based on the recognition between a DNA sequence and a protein. A new editing system based on DNA-RNA recognition was developed in 2013, which received the name of CRISPR/Cas [28]. This technology supposed a revolution in the field of genome editing, which is now accessible to almost every laboratory worldwide.

The increased accessibility of CRISPR/Cas system makes it a powerful tool in many research areas, from agriculture to ecological vector control or biomedicine. To the purpose of the present review, it is especially interesting to mention the broad applications of CRISPR/Cas system in biomedical research, ranging from targeted genome editing to the regulation of gene expression or even the labeling of endogenous sequences. This technology has a great potential to generate pre-clinical models of many human disorders, both in vitro and in vivo, that could help to understand the molecular pathways that lead to the development of a certain pathology [29,30,31].

### Fundamentals of Genomic Editing

**Figure 3:**
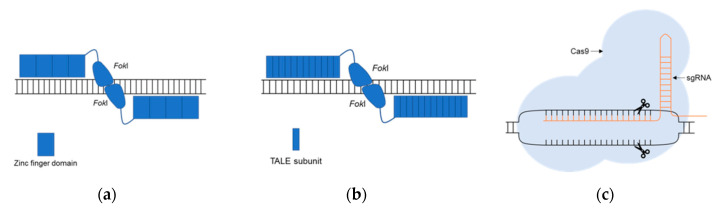
The main genomic editing systems available at the moment. (**a**) Zinc Finger Nucleases—ZFNs: two zinc finger nucleases act as a dimer, each one harboring a DNA binding domain and a DNA cleaving domain *Fok*I; (**b**) Transcription Activator-Like Effector Nucleases—TALENs: TALENs act as a dimer, each one harboring a DNA binding domain (TAL effectors) and a DNA cleaving domain *Fok*I; (**c**) CRISPR/Cas9: a sgRNA binds to the DNA and to the Cas9 endonuclease, facilitating the creation of double-strand breaks (DSBs) in the DNA. The image is original and was created by the authors of the present review.

All three systems (Figure 3) create specific breaks into the DNA, which in turn trigger the cellular DNA repair mechanisms. Eukaryotic cells have two main routes of DNA repair: non-homologous end joining (NHEJ) and homology-directed repair (HDR). NHEJ pathway is faster, but also prone to error, generating insertions or deletions (indels) due to its activity. NHEJ often alters gene’s reading frame or inserts stop codons at unusual places, generating truncated proteins that are unable to properly function. HDR pathway is more precise as it can correct alterations using a donor sequence as a template. Taking advantage of the HDR system allows the introduction of specific modifications in the genome, which can be as small as one single nucleotide [32].

ZFNs are a type of DNA-binding proteins that can be used to create double-strand breaks (DSBs) at desired positions in the genome. To function, this edition system requires two zinc finger nucleases, each one harboring two essential domains: a DNA binding domain and a DNA cleaving domain. The DNA binding domain is composed of protein modules, each one able to recognize a specific nucleotide triplet. The second essential domain of a ZFN is the sequence-independent cleaving domain, which is derived from the endonuclease *Fok*I (Figure 3a). The combination of both domains allows the ZFN to act as a site-specific nuclease [26,33]. ZFNs are an efficient editing system that can be applied to multiple experimental models, including cell cultures and animal models [34,35,36,37]. However, despite their efficiency, the use of ZFNs has not been widespread due to the difficulty of the experimental design and the required validation.

TALENs emerged in 2010 as an alternative to ZFNs. TALENs function is based on the combination of *Fok*I cleavage activity and transcription activator-like effectors (TALEs) (Figure 3b) which target individual base pairs. In comparison with ZFNs, TALENs are easier to synthesize, but the required protein design is still challenging [27,38].

As mentioned above, the most recently developed genomic editing system was CRISPR/Cas9 which is based on bacterial immune systems CRISPR type II. In comparison with ZFN and TALENs, CRISPR/Cas9 stands out for its relative simplicity, as it only needs two elements to function. The first one is the Cas9 nuclease, which contains two endonuclease domains, HNH and RuvC-like, which create DSBs in the DNA. The other essential element of this system is the single guide (sgRNA), which is composed of two regions: trans-activating CRISPR RNA (tracrRNA) and CRISPR RNA (crRNA). The tracrRNA, allows the binding between the Cas9 nuclease and the guide itself, whereas the crRNA is fundamental for the recognition of a specific target site in the genome (Figure 3c) [28,29,30,31].

The original model has been modified over the years, introducing modifications and improvements in its functioning. Nowadays, Cas9 can be substituted by other enzymes, expanding the applications of the technique.

CRISPR/Cas immune systems have been found in a wide range of prokaryotes, both bacteria and archaea. This indicates that there might be a broad number of Cas-like proteins that remain undiscovered to date, which could have new characteristics and/or properties of interest for genetic engineering purposes [39]. Some of them have already been characterized such as Cas13 family members, which are able to introduce breaks into RNA, opening the possibility of mRNA manipulation using the CRISPR system [40,41].

New types of Cas nucleases could be useful in order to broaden our battery of CRISPR/Cas modifying enzymes, but the possibility of engineering known nucleases, such as Cas9, is also interesting. For instance, a lot of effort has been put into the development of inducible forms of Cas9, as well as into altering its recognition site (PAM sequence) and improving its fidelity [42,43]. In addition, it is also intriguing the development of versions of Cas9 with one (Cas9 nickases, nCas9) or two (dead Cas9, dCas9) non-active catalytic domains. These modified Cas9s can be in turn fused with other enzymatic domains, which is the functional base of CRISPR interference (CRISPRi) [44], CRISPR activation (CRISPRa) [45], base editing [46] and prime editing [47].

One important drawback of CRISPR/Cas technology is the presence of off-target effects in the genome of the edited cells, which can be especially dangerous for clinical applications. Notwithstanding, there are mechanisms that can help to evaluate the occurrence of non-specific effects, such as whole-exome sequencing (WES) or whole-genome sequencing (WGS), although the latter generates a huge amount of data to be analyzed and biologically interpreted. Remarkably, the results of the studies carried to date seem to indicate that the occurrence of off-targets is, in fact, similar to the normal mutation rate of cells [48,49].

Genetic edition by CRISPR/Cas system has been applied successfully on many model organisms, including Caenorhabditis elegans [50], Drosophila melanogaster [51], zebrafish [52], rodents [53], and even primates [54]. CRISPR/Cas has also been used in human cell cultures, both of somatic [28] and embryonic cell lines [55].

Both the introduction of indels (knockout, KO) and specific genetic modifications (knock-in, KI) can be a powerful tool to model gene–base disorders, as it allows researchers to precisely study the association between genes or genetic variants and the development of an altered phenotype.

## 3. In Vitro Models of ASD: The Stem Cell Revolution

Cellular models are very useful for studying diseases with an important genetic contribution, especially if these diseases cause alterations in cell types easy to maintain in the laboratory. As it was previously mentioned, ASD often has a strong genetic component, and its effects are primarily seen on cells of the central nervous system associated with high cognitive functions. These cell types cannot be obtained from biopsies, which supposes an obstacle for the study of ASD bases using cell cultures as a model. In addition, neurons are a highly specialized cell type with a low proliferation rate, so they cannot be cultured for the long term, and thus, model cell lines are hard to establish [7,56,57].

However, this scenario changed in 2006, when Yamanaka and his collaborators identified mechanisms that allow reprogramming adult somatic cells representing new perspectives in molecular biology and biomedicine. These techniques allow the transformation of differentiated cell lines into induced pluripotent stem cells (hiPSCs) by expressing four genes, known as the Yamanaka factors (Oct3/4, Sox2, Klf4, c-Myc) [58]. The main advantages of hiPSCs are their self-renewal capability and their differentiation potential. A new and exciting possibility for the study of neurodevelopmental disorders was then born, as hiPSCs can afterwards be differentiated into cell types from the nervous system. The development of novel reprogramming methods and differentiation protocols makes it now possible to generate cell lines directly from patients, obtaining, as a result, specialized in vitro models to study the cause of the disorder in a particular individual [57,59].

Cellular models directly derived from patients have several advantages in comparison with other in vitro models, such as embryonic cell lines. With this approximation, models for disorders caused by rare variations can be created, which is the case for ASD (Table 3). Cellular models obtained from patients have proven to be highly robust, reliable and realistic, conserving the genetic background of the source. As they match the genetic background of the patients, the biological base of their respective disorders can be analyzed. An additional advantage of these cellular models is that they can be used to revert potentially pathogenic genetic variants, which can help to validate the association between the detected genotype and an altered phenotype [57,59,60].

Cell lines obtained from patients are versatile models, in which analyses to establish the cell and molecular mechanism implied in the curse of the disorder, can be conducted. When addressing neurological disorders, it is possible to study alterations in neuronal morphology, synaptic transmission, cell migration and differentiation capability, among others [56,59,60].

These models are useful to establish a relationship between a genotype and a phenotype, but also to develop new therapeutic approaches, including cell therapy and pharmacological treatments. This can be achieved by studies for the identification of new therapeutic targets or biomarkers, as well as drug sensibility assays, which are helpful to validate the action of the selected drugs prior to clinical assays [57,59].

For all the stated reasons, this approach opens new possibilities for the study of the molecular bases of complex disorders, such as ASD. Several research groups have been working in this field to study both syndromic and non-syndromic forms of ASD. In Table 3, a list of ASD-associated genes that have been studied using this approach can be found. Some long non-coding RNAs (lncRNAs), such as *PTCHD1-AS* or *COSMOC* [61,62] are also included. Further information about recent studies that implicate lncRNAs, other non-coding mutations, and regulatory variants in ASD susceptibility can be found in the excellent review by Ross et al. [63].

Despite the advantages of in vitro models, it is undeniable that cell culture cannot fully recapitulate all the complexity behind the development of ASD, for this reason, animal models are still a fundamental tool to fully understand them.

## 4. Animal Models in ASD Research

Traditionally, animal models have been used to study the complex background of ASD, as it was not possible to establish human neuronal cell cultures with an unlimited proliferation capability. Animal models are especially useful for studying disorders of the central nervous system because they help to validate the implication of selected genes in the curse of the disorder.

For an organism to be an adequate model for any human disease or disorder, including ASD, it should have the following characteristics: strong analogies to human phenotype (Table 4); the same biological alteration that causes the human disease; and analogous response to treatments that could ameliorate the human disease or disorder [7,93,94,95].

Two main approaches have been used to identify animal models for ASD. The first approach is forward genetics, in which ASD-like phenotypes are identified in the selected animal model, and then the molecular bases of the observed alterations are elucidated. The second approach is reverse genetics, in which targeted mutations are introduced into the genome of the animal model, and then the phenotype is characterized [96].

Rodents are the most used animal models in neuroscience research, being *Mus musculus* the most frequent one. This does not mean that mice are better models than other species, but it has more to do with a practical issue: the mouse genome was sequenced first and tools to manipulate it were developed faster. Nevertheless, nowadays, this information and tools are available for a wide range of model organisms, some of them with a lot of potential in ASD modeling, such as *Rattus norvegicus* or *Danio rerio*. This means that new animal models to study ASD might be developed in the near future [96,97,98,99].

### 4.1. Rodents and the Modelling of Human Disorders

Rodents have several characteristics that explain why they have been so widely used to model human disorders. First, they have a short generation time and, due to their small size and their social behavior, they can be easily maintained in an animal facility in the laboratory. Additionally, their genome has been sequenced, revealing a high similarity with humans. In addition to this, tools to modify the genomes of both species have been developed, as well as neurological, behavioral (Table 4) and pharmacological assays to evaluate the presence of ASD-like alterations [53,98].

It is undeniable that central nervous system organization is more complex in humans than in rodents. This complexity is reflected not only in the number of regions present in the brain, but also in the number of cells and connections, as well as their diversity. These differences are translated into cognitive and behavioral differences, so it is very unlikely that a rodent model can fully recapitulate the phenotype observed in ASD patients. However, pathways involved in ASD development can be studied in rodent models by using gene editing technologies which lead to the development of animals with phenotypes similar to the observed in humans [7,94]. Although both *Mus musculus* and *Rattus norvegicus* are rodents and as such share some common characteristics, there are also key differences between them in terms of physiology, behavior and pharmacological response that affect the type of information that can be obtained from each one.

In terms of physiology, there is a clear difference in body size and weight between both species. The small size of mice can be useful in drug development assays as a lower dose is needed to treat the animal. However, the bigger size of the rats can be an advantage if brain surgery is necessary or if imaging techniques are used, but it also increases the housing costs.

Concerning neurophysiology, some differences are notorious between mice and rat brains. First, it has been shown that some neurotransmitters and their receptors have different distributions on both species. Second, it also has been observed that both species have differences in their neurogenesis, affecting regions such as the hippocampus or the cortical regions, which have been associated with ASD development [97,98].

In terms of pharmacology, proteins derived from mice and rats are highly similar, but key substitutions in important regions to ligand binding have been identified. Is important to acknowledge these differences, especially when using these models to identify potential new drugs for ASD treatment, as they might not perform equally in humans [97,98].

With reference to behavior, both species live in hierarchical groups with complex social interactions, but the interaction between individuals is quite different in both cases. Mice are more territorial and aggressive than rats, but also less impulsive. There are also differences in their cognitive capacities, as rats are easier to train and perform more stably over time, not being as altered by the human presence as mice [97].

Regarding communication, both species have acoustic (USVs) and olfactory signals (pheromones) to transmit messages to conspecifics, but these are slightly more rich in *R. norvegicus*, with both adults and pups emitting a wider range of sounds in different types of situations, from isolation to play [98].

Rodents have been a fundamental preclinical tool to clarify the complex etiology of ASD, as well as to test new potential treatments before clinical trials. One of the reasons for such success is that they can recapitulate the core symptoms of ASD: impairments in social interaction, communication and presence of repetitive behaviors [94,98].

#### 4.1.1. *Mus Musculus* in ASD Research

**Figure 4:**
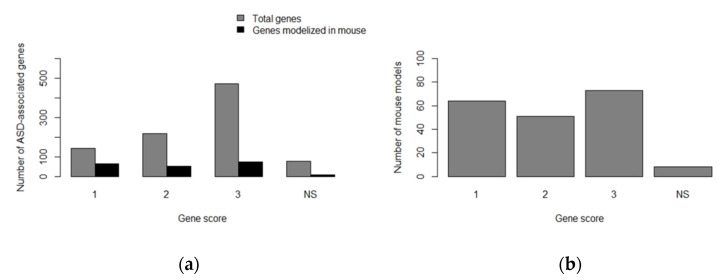
*Mus musculus* models developed to study ASD-associated genes. (**a**) Comparison between the human ASD-associated genes deposited in the SFARI Gene Database, and the number of ASD-associated genes modeled in *Mus musculus*. Genes are classified according to their SFARI gene score (NS = non-specified); (**b**) Number of mouse models developed to study ASD-associated genes, classified according to the SFARI gene score (NS = non-specified). The figure was elaborated using open-access data from SFARI Gene Database (obtained in January 2020) and R software [6,20,21].

Certain mouse strains with ASD-like phenotypes have arisen due to inbreeding procedures, for example, BTBR T+tf/J strain. This strain is very interesting as it recapitulates many of the human ASD-symptoms, such as social behavior impairments (reduced interaction between individuals, aversion for frontal interaction, etc.), communication impairments (altered patterns or responses to both USV and scent marking) and repetitive behaviors (increase in self-grooming, burying behaviors and preferences for certain objects or spaces). BTBR mice also develop difficulties in learning-related tasks and higher levels of anxiety in the presence of a menace. At the molecular level, this strain shows alterations in the development of the brain, which are also present in humans with ASD. Several ASD-linked genes have been identified to be disrupted in BTBR mice, such as kynurenine 3-hydroxylase, a protein involved in neuroprotection and dopamine signaling, Disc1, and Ext1, a protein involved in the synthesis of guidance molecules [94,100]. However, the majority of ASD relevant mouse models available to date have been generated using reverse genetics, by altering the orthologous ASD-linked genes in the mouse genome. Nowadays, there are nearly 200 mouse models (Figure 4) developed to study such genes, which can be found on SFARI GENE Database [20,21]. Examples of *M. musculus* models for ASD-candidate genes can be found in Table 5.

#### 4.1.2. *Rattus norvegicus* in ASD Research

Due to their more complex behavior and social interactions, rats have been postulated as a model organism with high potential to study NDDs, including ASD.

The first rat KO models available to study ASD were generated in 2010 using ZFN and ENU induced mutagenesis [98]. Nowadays, the number of available rat models has increased, including genetic models for certain ASD-risk genes (Table 6), and some pharmacological rescue models.

Nevertheless, despite the obvious suitability of rodent models for ASD modeling and the invaluable information they offer, there are still some noticeable drawbacks that have led researchers to opt for more manageable models, such as zebrafish.

### 4.2. Zebrafish and the Modeling of Human Disorders

In recent years, the zebrafish has been postulated as an ideal animal model for the study of the genetic background of several human diseases and remarkably, more than 800 laboratories around the world use nowadays zebrafish as a model [177]. The introduction of the zebrafish as an animal model dates back to the early 1960s, initially used to study vertebrate development and genetics [178]. Since then, researchers have progressively drawn on this animal in several human scientific fields, from genetic diseases, regeneration pathways or toxicology assays to high-throughput drug screenings [179].

Zebrafish is a freshwater fish, native from the streams of the south-eastern Himalayan region, and it owns its name due to its fusiform morphology and the horizontal stripes on each side of the body. There is a notorious sexual dimorphism, which allows the distinction between males and females [180]. Although this fish is able to survive in a range of temperatures from 12 °C to 39 °C in nature, its optimal temperature in controlled conditions is 28.5 °C [181,182]. The biological features that help to explain its use in laboratories, as well as its success as a translational model in biomedical research, in particular in neurosciences [96,99,183], have been increasingly listed since the 1990s [178]. It is worth highlighting the frequent reproduction (once a week), producing between 200 and 400 embryos per couple, enabling the performance of high-throughput assays. The external fertilization and optical transparency of embryos and larvae allow researchers to easily manipulate animals and observe their development, specifically imaging of neurodevelopmental processes and neural activity, even at a single-cell level without using invasive techniques [179]. In addition, zebrafish nearly completes basic development within 24 h, has rapid growth and sexual maturation (3–5 months), and interestingly, zebrafish has delayed development of the adaptive immune system (10–14 days), which is the main basis of its use in cancer research, and possesses an extraordinary tissue regeneration ability [184,185,186]. Furthermore, there are some other practical issues that make zebrafish stand out when compared with rodents, such as the relatively easy and cost-effective maintenance or the small size of adult individuals, which allows breeding a high number of animals in the facility.

#### 4.2.1. Zebrafish and Mammals: Conservation throughout Evolution

Comparative studies have revealed that the order of neurodevelopmental events across species is highly conserved, even also in zebrafish, although time points, complexity and organization differ, mainly regarding morphogenesis and neurogenesis. In this sense, morphogenesis of zebrafish brain is completed within 3 days and mechanisms behind the formation of different brain structures, such as the neural tube or the telencephalon, differ with respect to those in mammals [187,188,189]. Nevertheless, the most significant brain regions and major subdivisions, as well as cell types, differentiation, connectivity, signaling pathways and gene expression patterns, are highly conserved [190,191,192]. Additionally, there are some structural and functionally equivalent neuroanatomic regions such as zebrafish lateral, dorsal and medial pallium, which share characteristics with the human hippocampus, neocortex and amygdala, respectively [193]. While this review will not explain in depth zebrafish and mammals neural structures development and their conservation, we refer the reader to the excellent review by Kozol et al., 2018 [194].

Regarding structural homology and ASD, an interesting example of a critical period is the cerebellar structure and its development. In zebrafish, the cerebellar primordial becomes evident at 22 h post-fertilization (hpf) [189], and the differentiation of excitatory or inhibitory neurons, glutamatergic and GABAergic respectively, begins at 3 days post-fertilization (dpf) and layers are detectable at 5 dpf [195]. Equivalent to mammals, although in distinct expression domains, the expression by cerebellar progenitors of *atoh1* genes gives rise to the excitatory cells and the expression *ptf1a* leads to the formation of inhibitory cells [196]. Glutamatergic neurons include granule cells and GABAergic neurons include Purkinje cells and in the adult zebrafish such cells are arranged in three layers: molecular, Purkinje cell and granule layer [195]. Purkinje cells are fundamental for the cerebellar neural circuit and its function as they receive synaptic information, process it and relay such information through their efferent projections to the cerebellar nuclei which, in turn, connect the cerebellum to the brain and spinal cord, regulating several cognitive, language, motor, sensory and emotional functions [197]. It becomes then evident the importance that these cells have in the proper function of the nervous system and precisely, in the majority of ASD cases, one of the most reproducible and apparent observations is the significant reduction in Purkinje cells number and size [198,199,200]. Guissart et al., identified several mutations in a nuclear receptor (RORα), essential for cerebellar development, in families with variable neurodevelopmental delay and intellectual disability, including cognitive, motor and behavioral phenotypes. They developed a zebrafish mutant model by CRISPR/Cas9 and were able to recapitulate the neuroanatomical features of patients, showing a reduction of Purkinje and granule cells [201]. This is only an example that provides a rationale for using zebrafish as a model to study neurodevelopmental disorders such as ASD. Nevertheless, the specific role that Purkinje cells have in the development of ASD-like phenotypes is still unclear.

With regard to genetics, the zebrafish genome-sequencing project was initiated at the Welcome Trust Sanger Institute in 2001 and in 2013, Howe et al., released a high-quality sequence assembly of the zebrafish genome, showing that approximately 70% of the human genes have one zebrafish orthologue, being >80% human disease-related genes [202]. Regarding development, as mentioned before, expression patterns in early developmental genes are homologous in both zebrafish and humans and major neurotransmitter systems such as GABA, glutamate, norepinephrine, cholinergic and dopaminergic pathways as well as glial cells are conserved between both species [190,191,203,204]. In addition, Lovett-Barron et al. established a novel method to discover behavioral-related cellular elements and evidenced evolutionarily conserved cellular and molecular systems involved in basic neuromodulatory circuits [205].

In regards to behavior, it has also been demonstrated that zebrafish shares behavioral patterns with humans, including physiological, emotional and social responses [99].

Altogether, these data reaffirm the suitability of the zebrafish as a biomedical research model and its relevance to our understanding of genes, neural circuits and the physiopathology behind neurodevelopmental disorders as ASD.

Henceforth, we will focus on the available genetic strategies applicable in zebrafish in order to develop reliable models to functionally validate ASD-candidate genes, and the techniques that might be utilized to characterize morphological, molecular and behavioral features.

#### 4.2.2. Gene Targeting in Zebrafish

One of the main attractions of zebrafish as the disease-model animal is the relative ease and versatility to conduct genetic manipulations in embryos, from transient downregulation or overexpression of a certain gene to permanent gene-targeted mutations [52,206].

Regarding transient reverse genetic approaches, the most commonly used in zebrafish is morpholino-based (MO) expression silencing. MOs are small modified oligonucleotides that are able to bind a selected target by complementary knocking down the gene function without altering the sequence. MOs can either bind the translation start site of the mRNA and thus, interfere with the progression of the ribosomal initiation complex, or to the splicing sites of the pre-mRNAs, leading to abnormal mature mRNAs [207]. Since the release of these antisense oligos in the latest 1990s [208], and given their relatively low cost and ease of use, several zebrafish models have been developed in order to unravel the implication of specific genes in many human diseases. In Table 7, several examples of morpholino-based studies for ASD-candidate genes are shown. Nevertheless, despite its extended use in biomedical research and although the majority of zebrafish studies of neurodevelopmental disorder genes have been based on MOs, these molecules present important disadvantages that should be considered. Firstly, their transient effect (up to 4 dpf) do not allow to study the gene function beyond the early developmental stages [209]. In addition, it has been reported MOs may lead to off-target effects, resulting in non-specific phenotypes for the gene of study or triggering apoptosis through p53 pathway activation, so a careful design must be carried out, it is recommended to use a control MO, rescue experiments with RNA might be performed to confirm MO specificity and when possible, morphant phenotypes should be confirmed in genetic mutants [210,211].

With respect to the generation of stable zebrafish mutant lines, the Targeted Induced Local Lesions in Genomes (TILLING) has been largely used. This technique is based on the exposure to a mutagen known as ethylnitrosourea (ENU), an alkylating agent which, by ethylating oxygen or nitrogen atoms in DNA bases, induces error-prone replication and in turn, leading to random point mutations in the genome. Next, sequencing is performed in order to identify loss of function mutations. From the beginning of its use [212], this procedure has been successfully applied to generate several models of KO zebrafish. This methodology has been quite useful to correlate specific genes with observed phenotypes, although the generation of a stable mutant line for a gene of interest is relatively limited as it is difficult to identify the desired mutation, costs are substantial and screening zebrafish libraries takes a long time [213]. Some zebrafish ENU knockout models for ASD-candidate genes are listed in Table 7.

In order to solve TILLING drawbacks, nuclease-based technologies were later introduced, speeding up the zebrafish knockout generation and, as previously mentioned, these techniques include TALEN and ZFN, whose functioning is basically the same [214,215]. Despite both techniques enabled researchers to improve the generation of zebrafish mutant lines, it is challenging to specifically design such systems, there is a high ratio of off-target and they are still time and cost consuming. Examples of knockout zebrafish models for ASD-candidate genes are shown in Table 7.

Recently, due to the development and optimization of new genetic editing protocols based on CRISPR/Cas system more accurate mutant zebrafish lines were achieved, as the system offers superior efficiency and flexibility with respect to the previously mentioned gene-editing methods [52,216,217]. With regard to CRISPR and neurodevelopmental disorders and in order to highlight its large applicability and utility, it is worth mentioning the extraordinary study recently performed by Thyme et al. They focused on more than 100 genomic loci at which common variants exhibited genome-wide significant associations in a schizophrenia case/control analysis and performed high-throughput CRISPR/Cas9 (132 genes) in zebrafish. By doing so, they were able to observe and describe a phenotypic landscape of schizophrenia-associated genes, to prioritize more than 30 candidates and to provide hypotheses to associate specific genes with biological mechanisms [218]. In Table 7, some examples of CRISPR/Cas9 zebrafish models are listed.

Aside from these genome-editing techniques, several transgenic zebrafish lines fluorescently labeled have been developed throughout the last years, enabling researchers to better characterize neurodevelopmental zebrafish models. Table 8 summarizes some of the available transgenic lines and their specific expression pattern.

#### 4.2.3. Characterization of Zebrafish Models

Once the zebrafish knockdown or knockout model to study ASD-candidate genes is generated (with or without transgenic lines), there are several techniques that might be utilized to its accurate characterization, being mainly focused on morphological, molecular and behavioral features.

Regarding morphological characterization, the parameters to be analyzed may include a series of general observations such as body, heart, head, eyes otolith or jaw malformations, yolk deformation or edema and tail bending. Secondly, in order to determine if there exists a delay or abnormality in development some measures might be taken, such as body length, head, eye and yolk sac area or otolith–eye and jaw–eye distance, as well as the different brain regions thickness, area and weight [246,267,268]. This characterization is image-based and might be performed manually, or with available commercial image software.

To molecularly characterize zebrafish knockdown or knockdown embryos, researchers can draw upon several techniques, but some of the most commonly applied when it comes to functionally validate candidate genes in the zebrafish model are summarized below.

With regard to gene expression patterns, many of the genes mentioned in transgenic lines in Table 8 can serve as markers in qPCR assays, which offer information about how much the gene is expressed, or in in situ hybridization (ISH) assays with RNA probes, which allow localizing where the gene is being expressed in a precise time point. Other markers to perform ISH or qPCR with, that may be useful in neurodevelopment research are *sox2* (neural stem cells self-renewal and pluripotency cells), *vglut2.2* (glutamatergic marker), *th1* (dopaminergic marker), *neurog1* (neuronal determination marker), *c-fos* (neuronal activator marker), *crh* (paraventricular nucleus neurons), *c-myc* (tectal proliferation zone and retina), *emx1* (telencephalon) or *otx2a* and *pax2a* (diencephalon and midbrain–hindbrain boundary) [267,269,270]. In addition, immunofluorescence leads the researchers to know where the protein is acting, and if there are differences in the amount of protein among individuals, although these assays are relatively limited in zebrafish due to the absence of several specific antibodies. Nevertheless, some have been successfully used such as anti-serotonin (serotonergic neurons), anti-GFAP (radial glia cells) [267], acetylated anti-α-tubulin (brain axonal tracts), anti-sox10 (neural crest cells migration) [223], anti-homer1 (post-synaptic protein), anti- synaptophysin (pre-synaptic terminals) [246], znp-1 (primary motor neurons) [269], anti-phosphohistone H3 (M-phase, cell proliferation) [36,226], anti-PCNA (cell proliferation) [270], anti-caspase3 (apoptotic cells) [228] or anti-PSD95 (synaptic marker) [271].

Transcriptomic analyses may be performed in-depth with RNA-sequencing (RNA-seq), although it requires a great amount and high-quality material. Excellent research with RNA-seq, which in addition highlights the suitability of zebrafish to study the implication of environmental factors in ASD-risk, was performed by Lee et al. They exposed embryos to valproic acid—known to induce autism-like effects—and further performed RNA-seq, finding a direct correlation between zebrafish transcriptome and several ASD-associated genes [272]. This technique may also be useful to assess genetic compensation among individuals with phenotypic variability [273].

Concerning behavioral characterization, the precocious behaviors that embryo and larvae display [274] have led to the development of many tests that have proven to be valuable and accurate in zebrafish models. In this sense, different research groups have already study alterations in learning abilities [275], decision-making [276], sensorial capabilities [277,278], emotional responses [279,280] and social interactions [107,281,282], among others. These mechanisms are especially relevant when using zebrafish as a model for studying ASD, as many of these responses are altered in humans suffering from these disorders.

Finally, due to the possibility to use large numbers of the individual to test different drugs or chemicals and the ease of the delivery of the substance—diluted in water [283]—zebrafish has been proposed to conduct high-throughput screenings of neuroactive compounds. This approach would enable the identification of novel compounds with the potential to be used in new treatments for ASD and other NDDs, and additionally, allow the evaluation of their toxicity [284,285].

#### 4.2.4. Limitations of Zebrafish to Model Human Disorders

As stated throughout this section, not only can zebrafish be used to study the genetic bases of ASD, but also to highlight the relevance of environmental factors on autism-like phenotypes development [285]. Nevertheless, there are some drawbacks that should be considered when using zebrafish to study human diseases, mainly related to the retention of many duplicate genes due to the whole genome duplication [286]. This means that in some cases, researchers ought to study both genes at the same time. However, this issue might be overcome if the planning of projects is accurately carried out.

## 5. Future Challenges

The present review has been focused on the need of developing reliable models to study the complex genetic background of ASD. These models could be useful to improve our knowledge of the disorder and also to lead the way to the discovery of new potential treatments for patients.

In a disorder as complex as ASD, with individuals having such a diverse genetic background, the possibility of creating personalized models could be very useful in the clinic. Due to the accessibility of the genome editing technologies, such as CRISPR/Cas, it is now more feasible to consider the possibility of creating models that recapitulate the causal mutations detected on patients, and in turn determine which drug therapy is more adequate for each case, which represents one of the first steps towards personalized medicine.

Another interesting approach that has recently been postulated is the possibility of conducting direct reprogramming in vivo [287]. Basically, this technology could allow differentiating adult somatic cells into other cell types without the need for a hiPSC intermediate state. This methodology could be very interesting as a cell therapy option for many diseases and disorders. An imbalance of excitatory and inhibitory neuronal networks has been correlated with the presence of ASD and other psychiatric disorders, which potentially could be corrected with this technology. However, more data need to be reconnected to confirm that this correlation is indeed causal and that cell therapy could be an adequate therapy option.

### 5.1. In Vitro Modelling

Despite the potential of NDDs modeling using hiPSC-derived cell lines, there are still some issues that need to be addressed. First, it is important to further optimize reprogramming strategies, as the heterogeneity between hiPSCs colonies is still high. By doing this, it is expected to reduce the variation between cell lines and increase the reproducibility of the experiments. CRISPR/Cas technology could help to address this issue, as it makes it possible to create isogenic cell lines that genetically differ only in the edited position [57]. However, CRISPR/Cas technology has not proven to be highly efficient on hiPSCs, probably due to the protective effect of p53 pathway. This pathway triggers apoptosis when DNA damage is detected, including the DSBs caused by Cas9 [288,289]. Increasing the efficiency of edition and reducing possible off-target effects are other two important milestones to overcome in the future.

Together with deoptimization of the reprogramming and editing mechanisms to reduce technical variability, it would be also necessary to focus on differentiation, standardizing culture conditions to obtain cell lines with reduced variability among each other. Such reduction becomes an especially relevant issue when complex disorders or diseases are being studied, as multiple factors contribute to the global phenotype.

In order to guarantee patients’ health and security and unless these issues are properly addressed, researchers may avoid the use of hiPSC-derived cell lines in cell therapy. Additionally, the cost of this type of therapy would still be, nowadays, extremely limiting for its global application.

### 5.2. In Vitro Modelling

New animal mutant lines could be used to study the phenotypic alterations caused by genes associated with ASD, including behavioral, neuroanatomical and morphological features. In this sense, not only can they be useful to address the etiology of the disorder, but also to conduct drug-screening assays in order to identify compounds with the ability to rescue such altered phenotype and thus, offering a promising sign that they could also be effective in human clinical trials [95,99]. In this regard, zebrafish has been postulated as a promising model and, although it is undeniable that zebrafish assays are not enough to translate a compound to clinical trials, it may allow the development of relatively fast and cost-effective drug-screenings, accelerating the pre-screening selection of compounds which in turn, might be further tested in other animal models, such as rodents.

Most models developed to study ASD were designed to study monogenic disorders, which represent a small fraction of ASD cases, so the establishment of new models to study more complex ASD backgrounds is one of the challenges that need to be overcome in the future decades [7].

In addition to this, there are other challenges that need to be addressed. First, behavioral assays need to be improved to better characterize the animal model phenotype and its equivalence with human alterations. The second issue is the lack of genetic diversity in most part of the developed models, as they come from a lineage of inbred animals. For sure, this is a complication for assessing the variability and complexity of a disorder, as well as for testing new potential drug targets to alleviate its symptoms [7].

Animal research has been a source of many debates in the past decade, as there is public concern about the ethics of the use of animal models in science [290,291]. Critics argue that the biological differences between humans and other animals can mislead research investigations (approximately 90% of drugs that pass animal tests do not pass clinical trials) and that they could be substitute by in vitro models [292]. Although it is true that non-animal models have proven to be very useful for certain assays, to date there is no in vitro model that can fully show the complexity of functioning of a living creature [293]. Taking this complexity into account is essential to have a better understanding of biological processes and also to identify the side effects of potential drug treatments. For this reason, many health organizations worldwide still require animal testing before allowing new compounds to go into clinical trials.

However, this does not mean that the use of animals in research should be free of regulation and animal facilities should follow standard procedures to ensure the well-being of the animals. This is necessary from both the ethical and the scientific point of view as trustworthy results can only be obtained if animals are maintained in accurate, non-stressful conditions [290,291,294].

In order to improve the way animals are used in research, many organizations have published guidelines and recommendations to help designing experiments that minimize the use of animals without compromising the acquisition of quality data. Examples include the 3Rs of animal research principle (Reduce, Replace and Refine), as well as more detailed guidelines such as ARRIVE (Animals in Research: Reporting In Vivo Experiments) or PREPARE (Planning Research and Experimental Procedures on Animals: Recommendations for Excellence), which every scientist should take into account for their experiments [295,296].

## Figures and Tables

**Table 1:** Standard clinical criteria for the identification and diagnosis of ASD in the population according to the DSM-5 [1].

Clinical Diagnosis Criteria for ASD
Deficits in social communication and interaction
Restricted and repetitive patterns of behavior, interests, or activities Symptoms present during early development Presence of impairments in important areas of an individual’s functioning Symptoms are not better explained by other mental disorder

ASD: Autism Spectrum Disorders; DSM-5: the 5th edition of the Diagnostic and Statistical Manual of Mental Disorders.

**Table 2:** Therapeutic options available to treat ASD symptoms. Available therapeutic approaches can be classified into three groups: psychosocial therapies, pharmacology and complementary alternative medicine. In the table below, it can be found a list of the available therapies divided into these three categories, including a brief explanation on which ASD symptoms can be ameliorated by their use, as well as their previously reported side effects [24,25].

Type of Therapy	Therapy	Procedure	Areas with Improvement	Side Effects
Psychosocial therapies	Applied behavior analysis (ABA)	Repetition of learning trials (positive reinforcement)	Intellectual functioning, language, daily living skills and socialization	Long-term and costly therapy, need patient’s cooperation and motivation
	Pivotal Response Treatment (PRT)	Targets specific skills and motivations	Improve communication skills and less disruptive behaviors compared to ABA	No significant side effects
	Parent-mediated early interventions	Interventions that can be applied at home by parents	Socialization and communication	No significant side effects
	Social skills interventions	Interventions to improve social skills	Emotional regulation, communication and socialization	No significant side effects
Pharmacology	Risperidone	Atypical Antipsychotics	Irritability, socialization and communication	Weight gain, increased appetite and somnolence
	Aripiprazole	Atypical Antipsychotics	Irritability	Weight gain and somnolence
	Olanzapine	Atypical Antipsychotics	Irritability	Weight gain
	Ziprasidone	Atypical Antipsychotics	Irritability	Cardiovascular alterations and somnolence
	Paliperidone	Atypical Antipsychotics	Irritability	Weight gain and extrapyramidal symptoms
	Haloperidol	Typical Antipsychotics	Hyperactivity, stereotypical behaviors and learning on discrimination tasks	Somnolence, irritability and dystonic reactions
	Antidepressants: venlafaxine	Typical Antipsychotics	Repetitive behaviors, socialization and communication	Hyperactivity, inattention, nausea and polyuria
	Antidepressants: clomipramine	Typical Antipsychotics	Stereotypical behavior and anger management	No significant side effects
	Divalproex sodium	Mood stabilizers	Irritability and repetitive behaviors	No significant side effects
	Methylphenidate	Stimulants/atomoxetine/α-2 agonists	Hyperactivity	Appetite decrease, insomnia, irritability and emotional outbursts
	Atomoxetine	Stimulants/atomoxetine/α-2 agonists	Hyperactivity and impulsivity	No significant side effects
	α-2 agonists: clonidine and guanfacine	Stimulants/atomoxetine/α-2 agonists	Hyperactivity	Somnolence
	Naltrexone	Other medications	Hyperactivity and impulsivity	No significant side effects
Complementary alternative medicine	Melatonin		Sleep disturbances	No significant side effects

**Table 3:** Types of alterations observed in neural-like cell lines with a lack of expression of ASD-associated genes. Neural-like cell lines developed to study ASD have been obtained by the differentiation of human induced pluripotent stem cells (hiPSCs) from patients or by the inactivation of the selected ASD-associated gene in controls, using genomic editing systems.

Cell Lines Derived from hiPSCs	ASD-Associated Gene	Alterations Due to the Lack of Expression of ASD-Associated Gene	References
Cortical neurons	*EHMT1*	Reduced neurite length and complexity Altered neuronal activity Increased expression of proliferation genes Decreased expression of maturation and migration genes	[64]
	*MECP2*	Increased synaptogenesis and dendritic complexity Altered neuronal network synchronization	[65]
	*NRXN1*	Altered ion transport and calcium signaling	[66]
	*PTCHD1*	Decreased frequency of miniature excitatory postsynaptic currents N-methyl-D-aspartate receptor (NMDARs) hypofunction	[61]
	*PTCHD1-AS*	Decreased frequency of miniature excitatory postsynaptic currents	[61]
	*SHANK2*	Increased number of synapses, dendritic length and complexity Increased frequency of spontaneous excitatory postsynaptic currents Altered expression of genes associated to neuronal morphogenesis, plasticity and synapse	[67]
	*SHANK3*	Synaptic alteration and decreased dendritic spines	[68,69]
	*TSC2*	Mitochondria disorganization and altered mitophagy Increased soma size and neurite number mTORC1 signaling pathway hyperactivation Increased neuronal activity and upregulation of cell adhesion genes	[70,71]
Dopaminergic neurons	*RELN*	Altered neuronal migration	[72]
Glutamatergic neurons	*AFF2*	Alteration in genes associated with neuronal development Decreased synaptic activity: reduced spontaneous excitatory postsynaptic currents	[73]
	*ASTN2*	Alteration in genes associated with neuronal development Decreased synaptic activity: reduced spontaneous excitatory postsynaptic currents	[73]
	*ATRX*	Alteration in genes associated with neuronal development Decreased synaptic activity: reduced spontaneous excitatory postsynaptic currents	[73]
	*CNTN5*	Increased neuronal activity	[74]
	*KCNQ2*	Decreased synaptic activity: reduced spontaneous excitatory postsynaptic currents	[73]
	*SCN2A*	Alteration in genes associated with morphogenesis Decreased synaptic activity: reduced spontaneous excitatory postsynaptic currents	[73]
Neuron-like cells	*ARHGEF9*	Altered mTORC1 signaling pathway	[75]
	*CACNA1C*	Altered calcium signaling Altered differentiation of neurons from cortical layers Increased production of norepinephrine and dopamine Altered expression of tyrosine hydrolase	[76,77]
	*CDKL5*	Alterations in neuronal activity	[78]
	*CHD8*	Altered expression of genes associated with neural development, β-catenin/Wnt signaling, extracellular matrix and skeletal system development	[79]
	*COSMOC*	Impaired redox homeostasis Altered *PTBP2* splicing	[62]
	*FMR1*	Altered DNA methylation patterns Altered expression of genes associated with neuronal development, migration and maturation Altered neurite formation and neuronal differentiation	[80,81,82]
	*SHANK3*	Alterations in the soma and neurites, as well as alterations in synaptic transmission Altered expression of genes associated to motility and neurogenesis	[83,84]
	*TRPC6*	Reduce neurite length and complexity Altered glutamatergic synapse formation and reduced sodium influx	[85]
Neural organoids	*CHD8*	Alterations in the expression of gens associated with neurogenesis, β-catenin/Wnt signaling, neuronal differentiation and axonal guidance	[86]
Neural progenitor cells	*NRXN1* *RELN*	Alterations in neuronal adhesion and differentiation Overactivation of mTORC1 pathway	[87,88] [89]
	*TRPC6*	Altered calcium signaling and expression of genes involved in cell adhesion and neurite formation	[85]
	*ZNF804A*	Altered expression of pathways mediated by interferon-α 2	[90]
Olfactory placodal neurons	*SHANK3*	Decreased number of synapses Alterations during neural development in the soma and neurites	[91]
Purkinje cells	*TSC2*	Hypoexcitability and synaptic dysfunction mTORC1 pathway hyperactivation Altered neuronal differentiation	[92]

**Table 4:** Assays to evaluate the presence of ASD-like alterations in model organisms (rodents and zebrafish). The behavioral assays are focused on detecting alterations in the three core areas affected in ASD-patients: socialization, non-social patterns of behavior (including repetitive behavior, motor alterations and limited range of activities) and communication [93,98,99].

Areas of Interest	Behavioral Assays in Rodents	Behavioral Assays in Zebrafish
Socialization	Social approach task: time spent with an unknown individual compared to a new non-social object Social preference tests (affiliation and recognition): time expend with an unknown animal in comparison with a familiar one Free interaction test: time spent interacting with unknown individuals compared to the time spent doing other activities (e.g., exploring) Social interactions: presence of interactions such as sniffing, following, pushing each other, etc.	S Preference for conspecific individuals. Shoal formation: measure of the natation distance between individuals (nearest neighbor distance, farthest neighbor distance, average inter-individual distance, time spent inside the shoal and polarization). Social interactions: presence of behaviors such as approaching, circling, mouth opening, biting, chasing, etc.
Non-social patterns of behavior	Open field test: presence and duration of spontaneous motor stereotypies. Reversal learning tasks: these tests evaluate the capability of the individual to habituate to a new routine. A routine should be established for the animals (acquisition phase) before a new one is introduced (reversal phase). Range of interests: measure of the exploratory activity of the subject animal. Burying behavior: presence of digging behaviors.	Repetitive behavior: presence of repetitive patterns of locomotor activity. Inhibitory avoidance response: a two-chamber tank is set up, with one chamber harboring an attractive stimulus paired with and aversive response. The latency of the individuals to enter the chamber harboring the aversive response is measured.
Communication	Ultrasonic vocalizations (USV): reduced levels of USVs or non-usual patterns of acoustic communication have been observed in models for ASD, as well as altered patterns of response to them. Habituation and dishabituation to social odors: response to a change in a familiar odor for a new one.	Non-available

**Table 5:** Phenotype observed in *Mus musculus* models of ASD-associated genes. The table includes the some of the developed models to study the function and implication in ASD of genes classified with score 1 (high confidence) or gene score 2 (strong candidate) in the SFARI Gene database [20,21]. In the cases in which several models have been developed, the phenotype column only includes their common characteristics; LOF—loss of function, SVZ—subventricular zone, MGE—medial ganglionic eminence, KO—knockout.

ASD-Associated Gene/*Mus musculus*	Gene Modification Technique	Main Phenotypical Observations	Reference
*ADNP*/*Adnp*	KO by homologous recombination	Embryonic lethality (KO) Developmental delay Decreased neuronal survival Social and memory impairments	[101,102,103]
*ARID1B/Arid1b*	KO by CRISPR/Cas9 Conditional heterozygous KO by Cas9 (floxed allele)	Increased lethality Abnormal brain and heart development Decreased neuronal precursor proliferation and cortical thickness Anxiety and social interaction alterations Decreased cognitive flexibility	[104,105]
*ASH1L/Ash1l*	KO with gene trap vector, piggyBac or CRISPR/Cas9	Increased lethality and infertility Delayed eye development Reduced adiposity Altered immune response Reduced chromatin modification	[106,107,108]
*CHD2*/*Chd2*	Targeted KO with cassette Cre-flox Conditional LOF in interneurons	Growth delay and increased mortality Abnormal synaptic transmission Reduced number of neural precursors and interneurons Altered hippocampal morphology Decreased object recognition memory Decreased spatial working memory	[109,110]
*CHD8/Chd8*	Knockdown (shRNAs) KO by CRISPR/Cas9 or Cre-LoxP	Altered brain development, corticogenesis and differentiation of neural precursors Reduced density of the dendritic tree Decreased myelination Increased anxiety and altered sociability Increased repetitive behaviors Altered memory patterns	[111,112,113,114,115,116,117,118]
*CIC/Cic*	Conditional LOF in the neocortex, hippocampus and pallium	Altered hippocampal and cortical morphology Reduced number of postmitotic excitatory neurons of the forebrain Reduced dendritic complexity Reduced social interactions	[119]
*CNTNAP2/Cntnap2*	Targeted KO by gene replacement	Delayed growth Cortical disorganization in the brain Decreased levels of neuroreceptors Repetitive behaviors and seizures Impairments in social interactions	[120,121,122,123,124,125]
*GABRB3/Gabrb3*	Conditional LOF in endothelial cells Targeted KO	Altered brain morphology Reduced number of interneurons Reduced neuronal migration Decreased levels of GABA neurotransmitter Increased seizures, anxiety and depression Reduced social and tactile memory	[126,127,128,129,130]
*PTEN/Pten*	Conditional LOF in: forebrain gabaergic and dopaminergic neurons; secondary progenitors in the subpallium SVZ; Purkinje cells; dentate gyrus, hippocampus, cortex or ventricular zone of the MGE	Increased lethality Altered brain morphology Reduced number of interneurons Increased neuronal size and connectivity Impaired neuronal differentiation Altered synaptic function Increased apoptosis in brain cells Increased thickness in the cerebellum Decreased number of Purkinje cells Reduced coordination Reduced social memory	[131,132,133,134,135,136,137]
*RELN/Reln*	Spontaneous mutation	Altered morphology of the brain, cerebellum, cortex and olfactory bulb Reduced number of Purkinje cells Altered neuronal migration patterns Altered metabolism of neurotransmitters Impaired coordination Increased anxiety response levels	[138,139,140]
*SCN2A/Scn2a*	Targeted KO by gene interruption Conditional LOF in dorsal telencephalic excitatory neurons	Increased apoptosis and mortality Seizures and hyperactivity Increased rearing Reduced anxiety responses	[141,142,143]
*SHANK2*/*Shank2*	Conditional LOF in Purkinje cells Targeted KO	Altered synaptic currents Increased anxiety and hyperactivity Reduced coordination Increased repetitive behaviors Reduced social approach Decreased spatial learning and memory	[144,145,146,147,148]
*TAOK2/Taok2*	Targeted KO by Cre-LoxP	Abnormal brain morphology and spine density Reduced dendritic length and complexity Reduced cortical lamination and thickness Impaired memory of context	[149]
*TBR1/Tbr1*	Conditional LOF in neurons of cortical layer 6 and subplate Targeted KO by homologous recombination	Altered brain morphology Reduced neuronal connectivity Reduced number of interneurons Altered differentiation of brain cells Altered cortical organization Altered synaptic currents Increased anxiety aggressiveness Increased aggressive	[146,150,151,152,153]
*UPF3B/Upf3b*	Targeted KO by gene trap	Reduced spine density Altered morphology of cortical neurons Poor differentiation of neural progenitors Impaired sensorimotor gating Abnormal clasping reflex Abnormal sleep pattern Impaired startle response to acoustic stimuli	[154]

**Table 6:** Phenotype observed in *Rattus norvegicus* models of ASD-associated genes. The table includes the developed models to study the function and implication in ASD of genes classified with score 1 (high confidence) or gene score 2 (strong candidate) in the SFARI Gene database [20,21]. In the cases in which several models have been developed, the phenotype column only includes their common characteristics.

ASD-Associated Gene/*Rattus norvegicus*	Gene Modification Technique	Main Phenotypical Observations	Reference
*BCKDK/Bckdk*	KO by spontaneous mutation	Neuronal alterations Reduced protein phosphorylation Infertility Altered development	[155]
*CACNA1C/Cacna1c*	KO by ZFN	Altered social behavior and reduced USVs Increased perseverative behaviors	[156,157]
*CNTNAP2/Cntnap2*	KO by ZFN	Seizures Hyperactivity Altered audition and sleep routines	[158,159]
*CYFIP1/Cyfip1*	KO by CRISPR/Cas9	Neuronal alterations Altered behavioral flexibility in learning tasks	[160]
*FMR1/Fmr1*	KO by ZFN	Increased repetitive behaviors and social alterations. Altered sensorimotor gating Memory difficulties Neuronal alterations Altered auditory responses	[161,162,163]
*MECP2/Mecp2*	KO by ZFN	High mortality Malocclusion Neuronal alterations Hypoactivity Altered social interaction and speech responses. Memory alterations Decreased grip strength	[164,165]
*NLGN2/Nlgn2*	Overexpression in the hippocampus	Decreased response to new stimuli and aggressive behavior	[166]
*NLGN3/Nlgn3*	KO by ZFN	Increased repetitive behaviors Hyperactivity and altered sleep routines Decreased body weight Altered juvenile play behavior and startle response Altered sensorimotor gating	[162,167]
*NRXN1/Nrxn1*	KO by biallelic deletion	Hyperactivity Altered startle response Memory alterations	[168]
*PTEN/Pten*	Heterozygous KO by ZFN	Neuronal alterations	[169]
*SCN1A/Scn1a*	KO by ENU mutagenesis	Increased repetitive behaviors Hyperactivity and anxiety Learning and memory difficulties Motor alterations Reduced dopamine levels	[170]
*SHANK2/Shank2*	KO by ZFN	Alterations in social behavior Hyperactivity and increased repetitive behavior Memory alterations Neuronal alterations	[171]
*SHANK3/Shank3*	KO by ZFN	Alterations in social behavior Neuronal alterations	[172]
*TCF4/Tcf4*	KO by CRISPR/Cas9 and knockdown by shRNA in the prefrontal cortex	Altered electrophysiological properties in neurons	[173]
*TSC2/Tsc2*	KO by spontaneous mutation	Enhanced episodic-like memory Enhanced seizure-induced plasticity Increased induction of phospho-p42-MAPK in the hippocampus Increased basal oxygen consumption in the brain	[174,175]
*UBE3A/Ube3a*	KO by CRISPR/Cas9	Motor, learning and memory difficulties	[176]

**Table 7:** Phenotype observed in *Danio rerio* ASD-associated genes models. Genes are classified with score 1 (high confidence) or score 2 (strong candidate) following the SFARI Gene database [20,21]. In the cases in which several models have been developed, phenotype refers to the characteristics shared by all of them.

ASD-Linked Gene/*Danio rerio*	Gene Modification Technique	Main Phenotypical Observations	Reference
*ARID1B*/*arid1b*	Knockdown by MOs	Reduced body length Altered expression of chondrogenic/osteogenic genes	[219]
*ARX*/*arxa*	Knockdown by MOs	Altered brain development Neuronal alterations	[220]
*AUTS2*/*auts2a* and *auts2b*	Knockdown by MOs	Microcephaly Altered jaw development Motor alterations Neuronal alterations	[221]
*CACNA1C*/*cacna1c*	Knockdown by MOs	Cardiac alterations Altered jaw development	[222]
*CEP41*/*cep41*	Knockdown by MOs	Neuronal alterations Social behavior alterations	[223]
*CHD2*/*chd2*	Knockdown by MOs	Altered development Microcephaly, abnormal body curvature Swim bladder absence Motor difficulties	[224]
*CHD8*/*chd8*	Knockout by CRISPR/Cas9 and knockdown by MOs	Macrocephaly Reduction in post-mitotic enteric neurons	[225,226]
*CNTNAP2*/*cntnap2a* and *cntap2b*	Knockout by ZFN	Altered development Microcephaly Neuronal alterations Motor alterations	[36]
*CTNND2*/*ctnnd2b*	Knockdown by MOs	Reduced body length Notochord alterations	[227]
*DYRK1A*/*dyrk1a*	Knockout by TALENs	Altered response to social stimuli	[228]
*FMR*/*fmr1*	Knockout by ENU-mutagenesis and CRISPR/Cas9	Altered cephalic development Hyperactivity Increased anxiety Altered social behavior Learning difficulties	[229,230,231]
*KCNJ10*/*kcnj10*	Knockdown by MOs	Motor alterations Altered development	[232]
*KDM6A*/*kdm6a*	Knockdown by MOs	Reduced body length Altered development Notochord alterations Neuronal alterations	[233,234]
*MECP2*/*mecp2*	Knockout by ENU-mutagenesis and knockdown by MOs	Altered immune response Neuronal alterations	[235,236,237]
*MET*/*met*	Knockdown by MOs	High mortality Neuronal alterations	[238]
*MYT1L*/*mytl1a* and *mytl1b*	Knockdown by MOs	Reduced levels of oxytocin	[239]
*NBEA*/*nbea*	Knockout by ENU-mutagenesis and TALENs	Neuronal alterations Altered response to startle stimuli	[240]
*NR3C2*/*nr3c2*	Knockout by CRISPR/Cas9	Altered social behavior Altered sleep routines	[241]
*OXTR*/*oxtr*	Knockout by TALENs	Altered oxytocin signaling pathway Memory alterations in social and non-social recognition	[242]
*RELN*/*reln*	Knockout by TALENs	Altered social behavior Altered serotonin signaling pathway	[243]
*RERE*/*rerea* and *rereb*	Knockout by ENU-mutagenesis	Altered startle response to stimuli Vision and hearing difficulties	[244]
*SHANK3*/*shank3a* and *shankb*	Knockout by CRISPR/Cas9	Altered development Neuronal alterations Reduced social behavior, hypoactivity	[245,246]
*SYNGAP1*/*syngap1a* and *syngap1b*	Knockdown by MOs	Delayed development High mortality Neuronal alterations Motor difficulties	[245]

**Table 8:** Examples of developed zebrafish transgenic lines.

Transgenic Line	Expression Pattern	Reference
ath5:GFP	Retinal ganglion cells	[247]
brn3c:GFP	Retinal ganglion cells	[248]
dat:EGFP	Dopaminergic neurons	[249]
elavl3:lynTagRFP	Post-mitotic neurons	[250]
En-1:GFP	Circumferential ascending interneurons	[251]
flk1:GFP	Endothelial cells	[252]
gad1b:RFP	Gabaergic neurons	[253]
gfap:GFP	Radial glial cells	[254]
glyt2:GFP	Glycinergic neurons	[255]
gsx1:GFP	Gabaergic neurons	[253]
isl1:GFP	Cranial motor neurons	[256]
kctd15a:GFP	Torus lateralis	[257]
mnx1:GFP	Motor neurons	[258]
neurod:EGFP	Immature neurons	[259]
neurog1:GFP	Primary neurons	[260]
olig2:EGFP	Oligodendrocytes	[261]
pet1:GFP	Serotonergic neurons	[262]
qrfp:GFP	Rostral hipothalamus	[263]
sox10:GFP	Neural crest cells/Neurocranium cartilague	[264]
tbx2b:EGFP	Cone photoreceptor cells	[265]
Vglut2a:GFP	Glutamatergic neurons	[253]
vmat2:GFP	Monoaminergic neurons	[266]

## Abbreviations

ABA	Applied Behavior Analysis
*ADNP*/*Adnp*	Activity dependent neuroprotector homeobox
*AFF2*	AF4/FMR2 family, member 2
*ARHGEF9*	Cdc42 guanine nucleotide exchange factor 9
*ARID1B/Arid1b/arid1b*	AT-rich interaction domain 1B
ARRIVE	Animals in Research: Reporting In Vivo Experiments
*ARX/arxa*	Aristaless related homeobox
ASD	Autism Spectrum Disorders
*ASH1L/Ash1l*	ASH1 like histone lysine methyltransferase
*ASTN2*	Astrotactin 2
*atoh1*	Atonal bHLH transcription factor 1
*ath5*	Atonal bHLH transcription factor 7
*ATRX*	α thalassemia/mental retardation syndrome X-linked
*AUTS2/auts2a* and *auts2b*	Autism susceptibility candidate 2
*BCKDK/Bckdk*	Branched chain ketoacid dehydrogenase kinase
*brn3c*	POU class 4 homeobox 3
*CACNA1C/Cacna1c/cacna1c*	Calcium channel voltage-dependent, L type, α 1C subunit
Cas	CRISPR-associated genes
Cas13	CRISPR-associated endoribonuclease Cas13
Cas9	CRISPR associated endonuclease Cas9
*CDKL5*	Cyclin-dependent kinase-like 5
*CEP41/cep41*	Testis specific, 14
*CHD2/Chd2/chd2*	Chromodomain helicase DNA binding protein 2
*CHD8*/Chd8/*chd8*	Chromodomain helicase DNA binding protein 8
CIC/Cic	Capicua transcriptional repressor
c-Myc	MYC proto-oncogene
*CNTN5*	Contactin 5
*CNTNAP2/Cntnap2/cntnap2a* and *cntap2b*	Contactin associated protein-like 2
CNVs	Copy Number Variations
Cre	Cre recombinase
*crh*	Corticotropin releasing hormone
CRISPR	Clustered Regularly Interspaced Short Palindromic Repeats
CRISPRa	CRISPR activation
CRISPRi	CRISPR interference
crRNA	CRISPR RNA
*CTNND2/ctnnd2b*	Catenin (cadherin-associated protein), delta 2
*CYFIP1/Cyfip1*	Cytoplasmic FMR1 interacting protein 1
*D. rerio*	*Danio rerio*
*dat*	Dopamine transporter/Solute carrier family 6 member 3
dCas9	Catalytically dead Cas9
Disc1	Disrupted in schizophrenia 1
DNA	Deoxyribonucleic acid
dpf	Days post-fertilization
DSBs	Double-Strand Breaks
DSM-5	Diagnostic and statistical manual of mental disorders, 5th edition
*DYRK1A/dyrk1a*	Dual-specificity tyrosine-(Y)-phosphorylation regulated kinase 1A
*EHMT1*	Euchromatic histone-lysine N-methyltransferase 1
*elavl3*	ELAV like neuron-specific RNA binding protein 3
*emx1*	Empty spiracles homeobox 1
*En-1*	Engrailed homeobox 1
ENU	N-ethyl-N-nitrosourea
Ext1	Exostosin glycosyltransferase 1
*FMR1/Fmr1/fmr1*	Fragile X mental retardation 1
*Fok*I	Type IIS restriction endonuclease from *Flavobacterium okeanokoites*
GABA	γ-aminobutyric acid
*GABRB3/Gabrb3*	γ-aminobutyric acid type A receptor, subunit beta3
*gad1b*	Glutamate decarboxylase 1b
GFAP/*gfap*	Glial fibrillary acidic protein
*glyt2*	Sodium and chloride dependent glycine transporter 2
*gsx1*	GS homeobox 1
GWAS	Genome-Wide Association Studies
HDR	Homology-directed repair
hiPSCs	Human induced pluripotent stem cells
HNH	Endonuclease domain characterized by histidine and asparagine residues
hpf	Hours post-fertilization
Indel	Insertion and/or deletion
*isl1*	ISL LIM homeobox 1
*KCNJ10/kcnj10*	Potassium voltage-gated channel subfamily J, member 10
*KCNQ2*	Potassium voltage-gated channel subfamily Q, member 2
*kctd15a*	Potassium channel tetramerization domain containing 15a
*KDM6A/kdm6a*	Lysine demethylase 6A
KI	Knock-in
Klf4	Kruppel like factor 4
KO	Knockout
lncRNA	Long non-coding RNA
LOF	Loss of function
*M. musculus*	*Mus musculus*
MAPK	Mitogen-activated protein kinase
*MECP2/Mecp2/mecp2*	Methyl CpG binding protein 2
MET/met	Met proto-oncogene
MGE	Medial ganglionic eminence
*mnx1*	Motor neuron and pancreas homeobox 1
MOs	Morpholinos
mRNA	Messenger RNA
mTORC1	Mammalian target of rapamycin complex 1
*MYT1L/mytl1a* and *mytl1b*	Myelin transcription factor 1-like
*NBEA/nbea*	Neurobeachin
nCas9	Cas9 nickase
NDDs	Neurodevelopmental disorders
*neurod*	Neurogenic differentiation factor 1
*neurog1*	Neurogenin 1
NHEJ	Non-homologous end joining
*NLGN2/Nlgn2*	Neuroligin 2
*NLGN3/Nlgn3*	Neuroligin 3
NMDARs	N-methyl-D-aspartate receptors
*NR3C2/nr3c2*	Nuclear receptor subfamily 3, group C, member 2
*NRXN1/Nrxn1*	Neurexin 1
NS	Non specified
Oct3/4	Octamer binding transcription factor 3/4
*olig2*	Oligodendrocyte lineage transcription factor 2
*otx2a*	Orthodenticle homeobox 2a
*OXTR/oxtr*	Oxytocin receptor
p53	Tumor protein p53
PAM	Protospacer adjacent motif
PCNA	Proliferating cell nuclear antigen
PDD-NOS	Pervasive developmental disorder not otherwise specified
*pet1*	FEV transcription factor,
PREPARE	Planning Research and Experimental Procedures on Animals: Recommendations for Excellence
PRT	Pivotal Response Treatment
*PTCHD1*	Patched domain containing 1
*PTCHD1-AS*	PTCHD1 antisense RNA
*PTEN/Pten*	Phosphatase and tensin homolog
*ptf1a*	Pancreas associated transcription factor 1a
*R. norvegicus*	*Rattus norvegicus*
*RELN/Reln/reln*	Reelin
*RERE/rerea* and *rereb*	Arginine-glutamic acid dipeptide repeats
RNA	Ribonucleic acid
RNA-seq	RNA sequencing
RORα	Nuclear receptor ROR-α
RuvC	Endonuclease domain involved in DNA repair
*SCN1A/Scn1a*	Sodium channel, voltage-gated, type I, α subunit
*SCN2A/Scn2a*	Sodium channel, voltage-gated, type II, α subunit
SFARI	Simons Foundation Autism Research Initiative
sgRNA	Single guide RNA
*SHANK2/Shank2*	SH3 and multiple ankyrin repeat domains 2
*SHANK3/Shank3/shank3a* and *shankb*	SH3 and multiple ankyrin repeat domains 3
shRNA	Short hairpin RNA
*Sox2/sox2-sox2*	SRY-box transcription factor 2
*sox10*	SRY-box transcription factor 10
SVZ	Subventricular zone
*SYNGAP1/syngap1a* and *syngap1b*	Synaptic Ras GTPase activating protein 1
TALENs	Transcription Activator–Like Effector Nucleases
TALEs	Transcription Activator-Like Effectors
*TAOK2/Taok2*	TAO kinase 2
*TBR1/Tbr1*	T-box brain transcription factor 1
*tbx2b*	T-box transcription factor 2b
*TCF4/Tcf4*	Transcription factor 4
*th1*	Tyrosine hydroxylase 1
TILLING	Targeting Induced Local Lesions in Genomes
tracrRNA	Trans-activating crRNA
tRNA	Transfer ribonucleic acid
*TRPC6*	Transient receptor potential cation channel, subfamily C, member 6
*TSC2/Tsc2*	Tuberous sclerosis 2
*UBE3A/Ube3a*	Ubiquitin protein ligase E3A
*UPF3B/Upf3b*	UPF3B regulator of nonsense mediated mRNA decay
USVs	Ultrasonic vocalizations
*vglut2.2*	Vesicular glutamate transporter 2.2
*vglut2a*	Vesicular glutamate transporter 2.1
*vmat2*	Vesicular monoamine transporter 2
WES	Whole exome sequencing
WGS	Whole genome sequencing
ZFNs	Zinc Finger Nucleases
*ZNF804A*	Zinc finger protein 804A
